# PDbase: a database of Parkinson's Disease-related genes and genetic variation using substantia nigra ESTs

**DOI:** 10.1186/1471-2164-10-S3-S32

**Published:** 2009-12-03

**Authors:** Jin Ok Yang, Woo-Yeon Kim, So-Young Jeong, Jung-Hwa Oh, Sungwoong Jho, Jong Bhak, Nam-Soon Kim

**Affiliations:** 1 Korean Bioinformation Center (KOBIC), Korea Research Institute of Bioscience and Biotechnology (KRIBB), 111 Gwahangno, Yuseong-gu, Daejeon 305-806, Korea; 2 Laboratory of Human Genomics, Genome Research Center, Korea Research Institute of Bioscience and Biotechnology (KRIBB), 111 Gwahangno, Yuseong-gu, Daejeon 305-806, Korea

## Abstract

**Background:**

Parkinson's disease (PD) is one of the most common neurodegenerative disorders, clinically characterized by impaired motor function. Since the etiology of PD is diverse and complex, many researchers have created PD-related research resources. However, resources for brain and PD studies are still lacking. Therefore, we have constructed a database of PD-related gene and genetic variations using the substantia nigra (SN) in PD and normal tissues. In addition, we integrated PD-related information from several resources.

**Results:**

We collected the 6,130 SN expressed sequenced tags (ESTs) from brain SN normal tissues and PD patients SN tissues using full-cDNA library and normalized cDNA library construction methods from our previous study. The SN ESTs were clustered in 2,951 unigene clusters and assigned in 2,678 genes. We then found up-regulated 57 genes and down-regulated 48 genes by comparing normal and PD SN ESTs frequencies with over 0.9 cut-off probability of differential expression based on the *Audic and Claverie *method. In addition, we integrated disease-related information from public resources. To examine the characteristics of these PD-related genes, we analyzed alternative splicing events, single nucleotide polymorphism (SNP) markers located in the gene regions, repeat elements, gene regulation elements, and pathways and protein-protein interaction networks.

**Conclusion:**

We constructed the PDbase database to capture the PD-related gene, genetic variation, and functional elements. This database contains 2,698 PD-related genes through ESTs discovered from human normal and PD patients SN tissues, and through integrating several public resources. PDbase provides the mitochondrion proteins, microRNA gene regulation elements, single nucleotide polymorphisms (SNPs) markers within PD-related gene structures, repeat elements, and pathways and networks with protein-protein interaction information. The PDbase information can aid in understanding the causation of PD. It is available at http://bioportal.kobic.re.kr/PDbase/. Supplementary data is available at http://bioportal.kobic.re.kr/PDbase/suppl.jsp

## Background

The age-related neurodegenerative diseases prevalence is growing continuously due to a permanent increase in the human life span [1]. It affects almost half of all patients with dementia. Parkinson's disease (PD) is the second most common age-related neurodegenerative disease, which results in abnormalities in motor function [2]. Due to the high-frequency of PD, many researchers have tried to find the causation of PD. The disease is clinically characterized by impaired motor function, manifested by resting tremors, rigidity, bradykinesia, and postural instability [3]. PD is caused by the degeneration of dopaminergic neurons in the substantia nigra (SN) pars compacta [4].

Although the causation of PD is diverse and complex combination of the mitochondrial proteins' dysfunction, genetic variation effects in cell cycles, and environmental risk factors, it is now clear that genetic factors contribute to the pathogenesis of the disease [5-9]. However, the etiology of sporadic PD, occurring in 95% of the cases [9], is still not fully understood. To solve this problem, several resources have been incorporated to help PD studies such as MDPD [10] and PDGene [11]. MDPD provides a unique functionality to compare the differences in the type of mutations among ethnic groups manually examined by biomedical researchers [10]. PDGene at the Gene Prospector application provides evidence about human genes in relation to Parkinson's disease and risk factors from association studies [11]. Although useful integrated PD-related information has focused on genetic mutation and PD-association studies, there remains a limitation in public resource-dependent information. Therefore, we constructed experimental resources to investigate a wide spectrum of molecular events prior to integrating the PD-related public resources. Because public databases for SNPs and diseases are large, complicated, and difficult to use, we have developed the pipeline system to provide disease-related genes and genetic variations.

**Figure 1:**
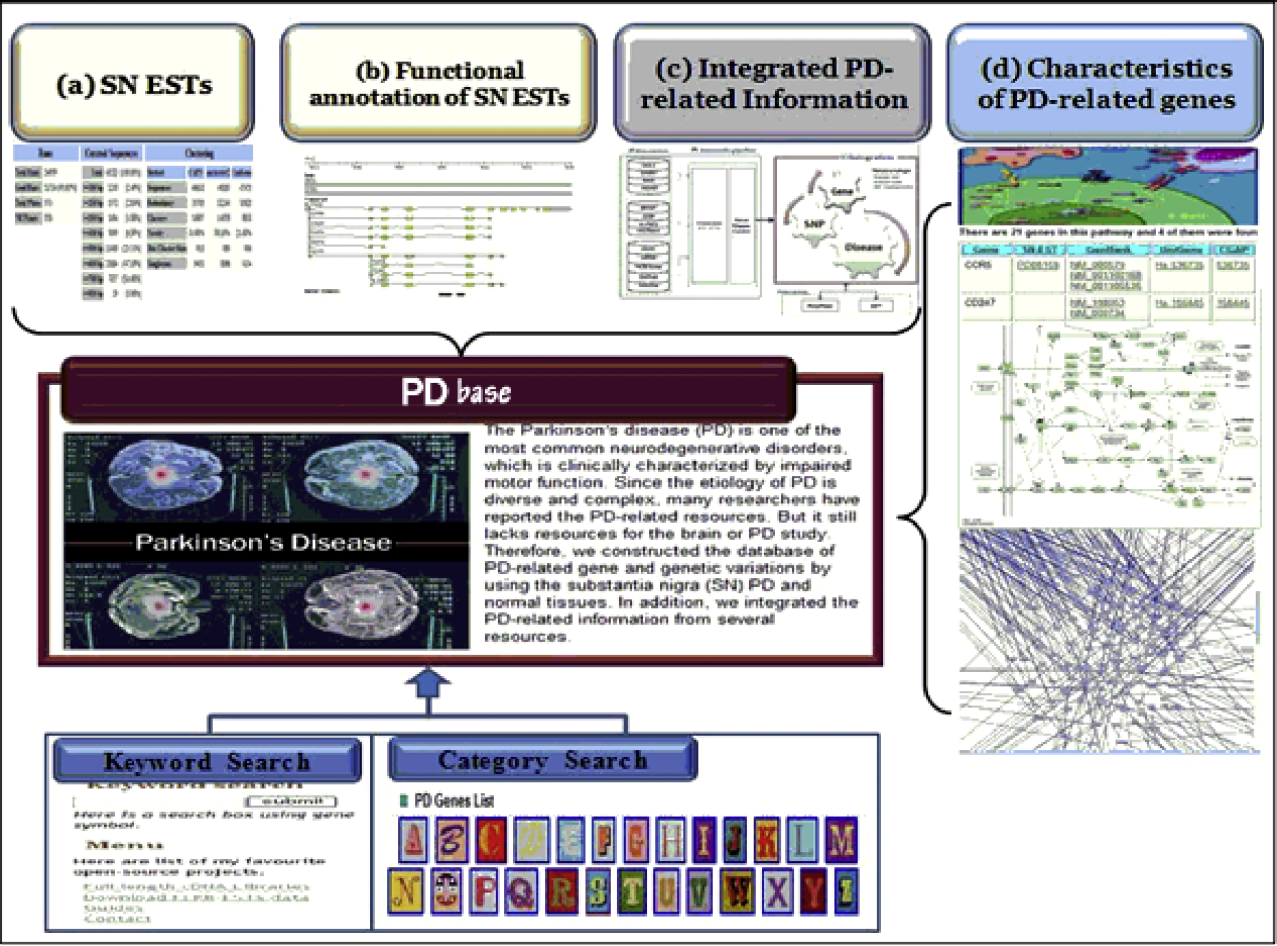
**Overview of the PDbase system**. PDbase contains four components: (a) statistics of the substantia nigra (SN) ESTs discovered from normal and PD patient tissues, (b) functional annotation of the SN ESTs showing alternative splicing events and the significant differences in expression between normal and PD SN, (c) integrated information containing genes and genetic variation related to Parkinson's disease from HGNC, UniProt, dbSNP, OMIM, HGMD, and GAD, (d) characteristics of the PD-related genes from microRNA elements, repeat elements, genes distribution on gene ontology, bio-pathways, and protein-protein interaction network.

Here, we report the PDbase database to capture characteristics of the general PD-related genes and abundantly expressed genes in SN (Figure 1). This database contains experimentally confirmed SN ESTs annotations and information about genes associated with PD: in detail, genetic variation, differential gene expression, gene-regulating elements, mitochondrial proteins, and pathways associated with PD-related genes. Users can explore the PD-related information according to the gene name or by clicking the gene list.

## Methods and results

### Substantia Nigra (SN) ESTs collection

We collected the 6,130 substantia nigra (SN) expressed sequenced tags (ESTs) from full-length cDNA libraries of brain SN normal tissues and PD patients SN tissues using oligo-capping methods in a previous study [3]. These SN ESTs were deposited in No.s DT214917~DT221046 at the dbEST database, NCBI. The full-length cDNA library was constructed using an improved capping method with the pCNS-D2 vector [12]. A normalized cDNA library was also constructed to obtain genes that are rarely expressed by the previous method [13]. We checked the repeat elements using RepeatMasker program http://repeatmasker.org. To get high-quality SN ESTs, we went through several filtering steps: 1) removing the short length ESTs, 2) removing ESTs contaminated by genomic DNAs and E. coli, and 3) removing ESTs not aligned in any UniGene cluster. We analyzed the PD candidate genes with this ESTs pool containing 2,850 SN ESTs from PD patients and 2,883 SN ESTs from normal tissues. We carried out the annotation of SN ESTs based on UniGene clusters and then obtained 2,679 genes' information with 5,733 UniGene clusters.

### SN ESTs clustering and expression

The annotation of the SN ESTs was carried out using the human RefSeq mRNA [14] and the UniGene database (build #217) for similarity comparisons based on the UniGene clusters (Shown in supplementary Data Table1). Our SN ESTs were clustered in 2,951 unigene clusters and assigned in 2,678 genes. Since we constructed the full-length cDNA libraries using the oligo-capping technique [15], these SN ESTs can be resourced to examine the multiple transcription start sites comparing mRNA transcription start sites. To investigate amino acid changes, we compared the SN ESTs sequences to the RefSeq protein sequences using BLASTX [16].

To study the global expression of genes possibly associated with Sporadic PD constituting most PD cases [17], we accounted for the number of SN ESTs from PD patients and normal tissues assigned in same gene. The frequency of each gene was analyzed by dividing the number of ESTs of a gene by the number of total clones merged into the UniGene database build #217 in each full-length cDNA library. Genes that were abundantly expressed were selected and listed among the ESTs. Significant differences in gene expression among the datasets were calculated using the *Audic and Claverie *method [18]. We analyzed the probability of differential expression between the normal full-length SN library and the PD full-length SN library at a cut-off probability of 0.9 (shown in supplementary Data Table2). Finally, we found 57 up-regulated genes and 48 down-regulated genes through the comparison of normal and PD SN ESTs frequencies. *MBP *of them was reported to be up-regulated in PD SN [19]. The up-regulated genes were associated with structural constituents of the myelin sheath, cytokine activity, transcription regulator activity, GTPase activity, calcium ion binding, or RNA binding on molecular function. The down-regulated genes were associated with oxidoreductase activity, serin-type endopeptidase inhibitor activity, phosphatidylethanolamine binding, mu-type opioid receptor binding, Rho GTPase activator activity, integrin binding, monooxygenase activity, or lipid binding.

### Genomic mapping of expressed sequence clusters

To create a consensus sequence, we mapped the SN ESTs, mRNAs, and UniGene EST clusters having at least one mRNA to exclude the pseudo genes onto human genome using BLAT and SIM4. We used consensus sequences to eliminate non-consensus features of each UniGene cluster, after filtering out EST sequencing errors or contamination by a minority of similar but paralogous sequences. Then EST-mRNA alignment was generated using the SIM4 program, producing a consensus sequence that excludes minority features such as unaligned ends and inserts due to chimeric sequences or unspliced introns. The matching genomic region was aligned with the complete set of ESTs and mRNAs for the UniGene cluster using BLAT [20] and SIM4 [21]. The SN EST sequences were aligned in human genomic sequences with a 75% minimum score and 90% minimum identity. When coordinates had non-canonical splice sites, we confirmed the exon-intron junction sites with the SIM4 program to perform alignments of expressed and genomic DNA sequence data efficiently and accurately, allowing for introns in the genomic sequence, and a relatively small number of sequencing errors [21].

### Alternative splicing analysis of SN ESTs

Alternative splicing was detected by a computational procedure using genomic-EST-mRNA multiple sequence alignments. Alternative splicing types were derived from these isoforms retaining all possible alternative splicing information [22]. SN ESTs with poor coverage were filtered out to remove non-consensus splice sites and regions with poor coverage. We categorized the alternative splicing types such as alternative start, alternative end, alternative 5' exon, alternative 3' exon, exon skipping, mutually excluded exons, or intron retention. Alternative starts and ends were identified if the first or last exon in a gene model was part of an alternative region. Alternative cassettes were labelled as such if the junction skipped one exon.

PDbase contains transcripts representing several alternative splicing events (Shown in Supplementary data, Table. 3). SN ESTs were associated with alternative splicing events in 321 genes. To examine candidate genes having the PD-specific alternative splicing patterns, we compared the alternative splicing patterns of normal SN ESTs and PD patients ESTs. We found that thirty-five PD-specific candidate genes having alternative splicing events were up-regulated in PD SN tissues: for example, *AQP1*, *DCXR*, *DKK3*, *EEF1A1*, *GNAS*, *PGK1*, *SUCLG1*, and *THTPA*. The major alternative splicing events in genes up-regulated in PD SN tissues are alternative transcription start or end sites. This may be a reason to construct SN full-length cDNA libraries using the oligo-capping method to replaces the cap structure specific to the 5' end of eukaryotic mRNA with oligonucleotides [12].

### Functional annotation

To provide the global PD-related gene features, we integrated PD-related gene information, as well as knowledge-based information. Because public databases for SNPs and diseases are large, complicated, and difficult to use, their integration is challenging. We collected 2,701 genes associated with PD and average 323 genetic variations in genomic region through our pipeline system for the disease-related gene and genetic integration [23]. This integrated information is based on human gene nomenclature (HGNC) [24] and UniProt [25], genetic variation from dbSNP (version 129) [26], and disease information from Online Mendelian Inheritance in Man (OMIM) [27], Human Gene Mutation Database (HGMD) [28], and the Genetic Association database (GAD) [29]. We examined the PD-related gene distribution to cover several domains of molecular and cellular biology based on the Gene Ontology database [30]. In addition, we surveyed the protein-protein interaction (PPI) of the PD-related genes from the Human Protein Reference Database (HPRD) [31]. It has been reported that the degeneration of dopaminergic neurons of SNpc is conducted by dysfunction of the mitochondrial complex through activation of mitochondria-dependent apoptotic molecular pathways [5]. Hence, we investigated mitochondrion proteins associated with PD from MitoDat (Mendelian Inheritance and the Mitochondrion) [32]. We found 31 mitochondrial proteins located in inner membrane (68%), outer membrane (19%), inter membrane space (6%), or matrix (6%). The numbers in parentheses indicate the percentages of mitochondrial proteins located in each organelle among the total number of mitochondrial proteins. There are, for example, solute carrier family 25 (*ANT1*, *ANT2*, *ANT3*), ATP synthase, H+ transporting, mitochondrial F1 complex (*ATP5A1*, *ATP5B*, *ATP5C1*, *ATP5F1*), and kinesin heavy chain member (*HK2*).

We also investigated molecular and cellular signalling pathways associated with PD-related genes from the BioCarta models http://www.biocarta.com/genes/index.asp and the KEGG databases [33]. To examine the RNA elements involved in the regulation of PD-related genes, we searched microRNA elements related to PD genes from the mirBASE database as experimental micro-RNA resources [34] and conserved mammalian microRNA regulatory target sites for conserved microRNA families in the 3'UTR regions of RefSeq genes predicted by TargetScanS at the UCSC table track [35]. In addition, we utilized multiple transcription start sites, CpG island, and repeat elements on the UCSC table tracks (download March 2009).

## Web interface

**Figure 2:**
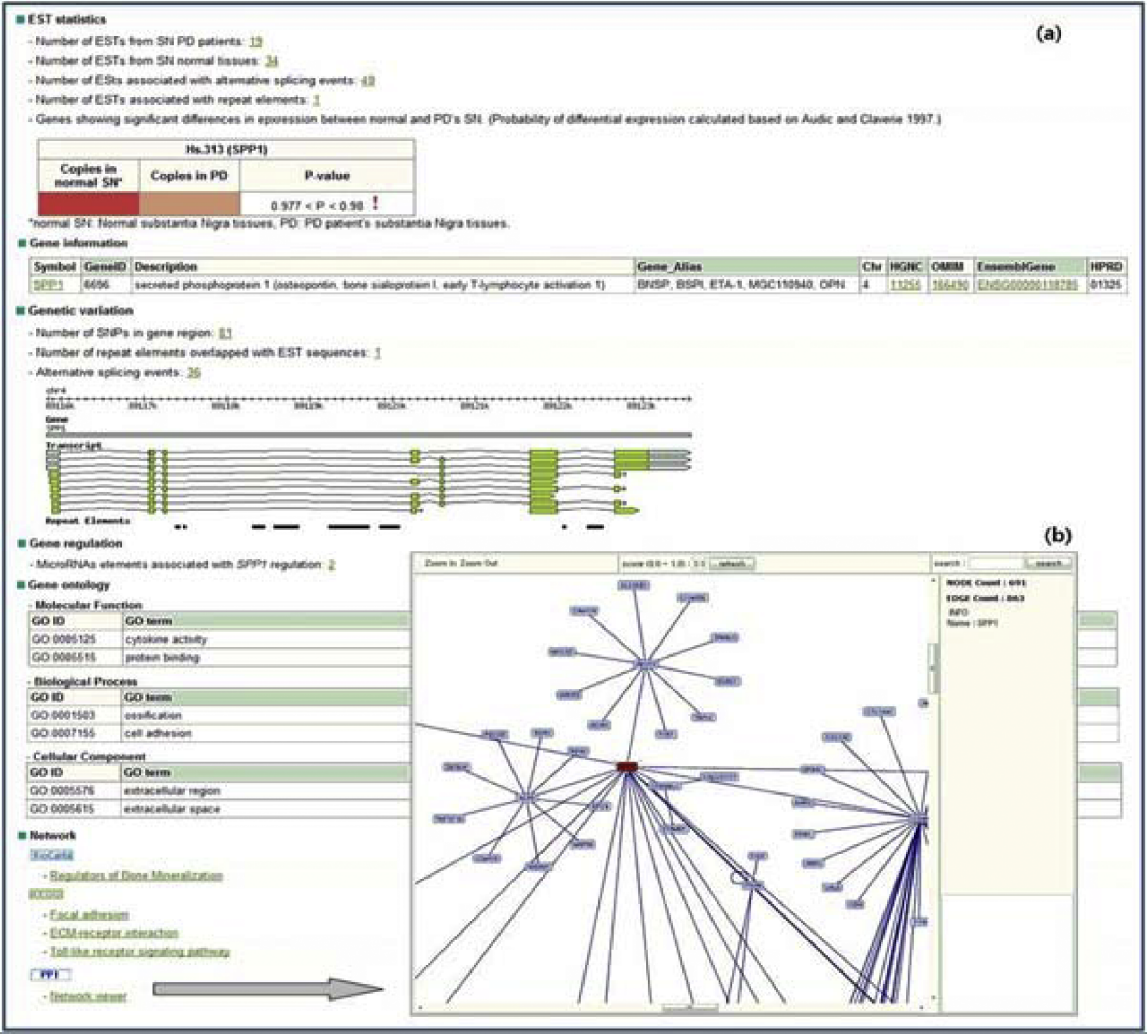
**Web interface of PDbase**. PDbase provides user-friendly web interfaces. When a user selects the '*SPP1*' gene from the category search page, search results (a) containing as follows: (1) "EST statistics" part: number of SN ESTs from normal and PD patients, number of SN ESTs associated with repeat elements and alternative splicing events, and probability of differential gene expression with SN ESTs. (2) "Gene Information" part: gene-related information about gene symbol and synonymous, description, HGNC, OMIM, Ensemble Gene, and HPRD. When click each link, user can get more detailed information. (3) "Genomic variation" part: detailed information about alternative splicing events, repeat elements, and SNPs in gene region annotated from SN ESTs. (4) "Gene Regulation" part: micro RNA elements to regulate the query gene (*SPP1*). (5) "Gene Ontology" part: gene distribution in three gene ontology categories. GO ids, terms, and evidence are based on the Gene Ontology database. (6) "Network" part: the query gene-related protein-protein interaction and biological pathways, such as BioCarta and KEGG. When the user clicks on the PPI viewer in a search result page, interactive java applet viewer (b) shows the interaction network containing the selected protein and its interacting partners.

The PD database server was developed in JAVA and Java Server Pages connected to MySQL. The main web interface allows exploration of integrated PD-related information through an alphabet ordered genes list and through query searching. The user can look at all the PD-related information at once. When the user clicks on a letter of the alphabet, the total PD-related gene list starting with the chosen letter can be seen. The user can then directly obtain results consisting of SN EST statistics, targeted gene information (name, synonyms, and title), genetic variation (SNP, alternative splicing events, and repeat elements), gene regulation (microRNA elements), gene distribution on the Gene ontology, and Network information such as pathways and PPI (Figure 2). When the user submits a gene, the system will return a list of genes related to the query along with gene aliases and types. When selecting a specific gene, PDbase shows the SN EST distribution, gene and genetic variants through the genome browser [36], and provides information on network properties of the PDbase through the PPI viewer [37]. These genetic variation and gene regulation elements displayed in the genome browser or PPI viewer facilitate the recognition of the disease-causing gene characteristics, especially if the SNP or gene regulation factors lie in the promoter region or in an intronic sequence [38].

To show an example of a PD-related gene search, we present query results for a gene, *SPP1*, which is secreted phosphoprotein 1 (osteopontin, bone sialoprotein I, early T-lymphocyte activation 1). When a user queries this gene, *SPP1*, comprehensive information including frequency differences between the two full-length libraries from SN PD and normal tissues is seen. There are 19 SN PD ESTs and 34 SN normal ESTs in the PDbase database. The *SPP1 *was down-regulated at a statistically significant level in more than one sample having a probability of 0.977 < p < 0.98. This gene is known as a high anti-apoptotic gene from a previous cell death activity study [3]. In addition, the user can get general gene information and genetic information containing the SNP marks located in this gene region, repeat elements, and alternative splicing events from the PD and normal SN ESTs. Three micro-RNAs can be associated with regulation of this gene, which has experimentally confirmed protein-protein interaction with eighteen other proteins and belongs to the regulators of the bone mineralization pathway. This SPP1 gene was represented as a PD target gene through our human SN ESTs analysis and verified using RT-PCR and neurotoxin, a 1-methyl-4-phenyl-1,2,3,6-tetrahydropiridine (MPTP)-treated mice model [3]. The query results through the PDbase database are more helpful to researchers than results obtained from published previous databases.

## Conclusion

We constructed a database of PD-related genes and genetic variation using SN ESTs, called PDbase. PDbase contains 2,698 genes and the biological characteristics of these genes in two ways: 1) through 303 cDNA libraries from human normal and PD's SN tissues and 2) by integrating information on disease-related genes and genetic variation. Mitochondrial DNA variants in PD play various roles. Mitochondrial dysfunction has been reported as the etiology of neurodegenerative diseases [39]. Thus, PDbase also provides the PD-related mitochondrion proteins, microRNA, Single Nucleotide Polymorphisms (SNPs) markers within PD-related gene structures, repeat elements, and pathways and networks with protein-protein interaction information. PDbase integrates not only public resources, but also un-reported PD target genes discovered from normal and PD SN ESTs. It can serve as specific biomarkers for PD or neurodegenerative diseases and novel drug development. Also, PDbase can provide insight into the pathogenesis of PD and identify molecular targets of potential therapeutic significance for the neurodegeneration.

## Availability and requirements

PDbase is freely available at http://bioportal.kobic.re.kr/PDbase/. All generated PD-related gene lists can be found at http://diseasome.kobic.re.kr/. The dynamic protein-protein interaction viewer based on Java applet technology requires Java-enabled web browsers.

## Competing interests

The authors declare that they have no competing interests.

## Authors' contributions

JOY constructed the database, coordinated this project, and wrote the manuscript. WYK developed the website and helped with database construction. SYJ and JHO constructed the full-length SN cDNA libraries and EST generation. SWJ developed the protein-protein interaction viewer. JB and NSK directed the project and supervised manuscript writing.

## Note

Other papers from the meeting have been published as part of *BMC Bioinformatics *Volume 10 Supplement 15, 2009: Eighth International Conference on Bioinformatics (InCoB2009): Bioinformatics, available online at http://www.biomedcentral.com/1471-2105/10?issue=S15.
